# Reinforcement Learning-Based Data Forwarding in Underwater Wireless Sensor Networks with Passive Mobility

**DOI:** 10.3390/s19020256

**Published:** 2019-01-10

**Authors:** Haotian Chang, Jing Feng, Chaofan Duan

**Affiliations:** Institute of Meteorology and Oceanography, National University of Defense Technology, Nanjing 211101, China; Haotian.Chang@anu.edu.au (H.C.); chaofanduan0926@163.com (C.D.)

**Keywords:** underwater wireless sensor networks, data forwarding, value of information, energy consumption, passive mobility, reinforcement learning

## Abstract

Data forwarding for underwater wireless sensor networks has drawn large attention in the past decade. Due to the harsh underwater environments for communication, a major challenge of Underwater Wireless Sensor Networks (UWSNs) is the timeliness. Furthermore, underwater sensor nodes are energy constrained, so network lifetime is another obstruction. Additionally, the passive mobility of underwater sensors causes dynamical topology change of underwater networks. It is significant to consider the timeliness and energy consumption of data forwarding in UWSNs, along with the passive mobility of sensor nodes. In this paper, we first formulate the problem of data forwarding, by jointly considering timeliness and energy consumption under a passive mobility model for underwater wireless sensor networks. We then propose a reinforcement learning-based method for the problem. We finally evaluate the performance of the proposed method through simulations. Simulation results demonstrate the validity of the proposed method. Our method outperforms the benchmark protocols in both timeliness and energy efficiency. More specifically, our method gains 83.35% more value of information and saves up to 75.21% energy compared with a classic lifetime-extended routing protocol (QELAR).

## 1. Introduction

Nowadays, marine surveillance, water contamination detection and monitoring, and oceanographic data collection are indispensable to the exploration, protection and exploitation of aquatic environment [1]. Because of the huge amount of unexploited resources in the ocean, there is an urgent need for research in the field of sensors and sensor networks [2]. Underwater Wireless Sensor Networks (UWSNs) has become a main approach to gain information from previously inaccessible waters. Traditional wireless sensor networks (WSNs) consist of a large number of sensor nodes randomly distributed in a detection field, and these nodes are usually either stationary or moving in limited ranges. However, in many practical scenarios, the movement of nodes is relatively large, such as nodes in UWSNs, delay-tolerant networks, vehicular networks, etc. Nodes in UWSNs can be categorized as stationary nodes and moving nodes. Stationary nodes are anchored to the water bottom while moving nodes can move in a preset velocity, such as Autonomous Underwater Vehicles (AUVs). Nevertheless, only a few researchers take passive mobility of nodes into account. More specifically, nodes may move along internal currents or vortices. Underwater nodes have no access to GPS signals, and the network topology is completely time varying due to irregular mobilities of water currents, which is essentially different from terrestrial WSNs. Meanwhile, due to dynamic topology changes and poor communication conditions underwater, data packets cannot be delivered to the sink nodes deployed on the water surface rapidly.

A major challenge of UWSNs is real-time requirements. For instance, fishery surveillance and real-time monitoring of precious assets such as petroleum pipelines. Specifically, report delay of sea properties such as temperatures may lead to serious loss of temperature sensitive sea animals, e.g., sea cucumbers, because they dissolve fast in high temperatures. Moreover, the detection of leakages of coal oil in early stage prevents water contamination and further resource waste. Therefore, we adopt the concept of the value of information (VoI) which evaluates information in terms of timeliness [3]. Additionally, UWSNs are energy constrained due to the fact that they cannot be recharged or replaced, so their ability to route data diminishes when sensor nodes run out of energy. Network lifetime remains the performance bottleneck which perhaps is one main obstacle in the wide scale deployment of wireless sensor networks [4,5]. In this case, energy consumption is also a fundamental issue in UWSNs.

In conclusion, it is significant to consider the timeliness and energy consumption of data forwarding in UWSNs, along with the passive mobility of sensor nodes. Motivated by the timeliness demand and the energy constraint of UWSNs, we aim to explore data forwarding in UWSNs with passive mobility, jointly considering the timeliness of packets and the energy consumption of the sensor nodes. Due to irregular dynamics of water, the node movement is unpredictable, i.e., the future status has little relevance to its historical trajectories. Consequently, the determination of the relay node of a sensor node depends on its current status and its neighborhood relationship. A reinforcement learning method is proposed in this paper. To the best of knowledge, we are the first to jointly consider timeliness and energy consumption of data forwarding in UWSNs with passive mobility.

The main contributions of this paper are as follows. We first formulate the problem of data forwarding, by jointly considering timeliness and energy consumption under a novel passive mobility model for UWSNs. We then propose a reinforcement learning-based method for the problem. We finally evaluate the performance of the proposed method through simulations. Experimental results demonstrated the validity of the proposed method and they also demonstrated the efficiency, compared with two benchmark methods.

The rest of this paper is organized as follows. Section 2 will review the related work of the proposed method. Section 3 will introduce the preliminaries, including the system model, notations and problem definitions, and the proposed method. Section 4 will show the simulation results. Section 5 will present the discussion of the simulation results and the look out for future work.

## 2. Related Work

Data forwarding for underwater wireless sensor networks has drawn a lot of attention in the past decade. There are several kinds of routing protocols that aim to improve energy efficiency, timeliness and adaptability to node mobility of UWSNs. In this section, we review the related work on this topic.

Lloret et al. have pointed out the urgent need and significance of UWSNs [1,2]. To satisfy the demand of timeliness of UWSNs, a lot of research was dedicated to decreasing the latency of data forwarding. Bassagni et al. [6] devised a forwarding method named Multi-modAl Reinforcement Learning-based RoutINg (MARLIN) protocol. The MARLIN strategy selects the best relay node along with the best communication channel, and it can be configured to seek reliable routes to the final destination, or to provide faster packet delivery. Gjanci et al. [3] proposed a Greedy and Adaptive AUV Path-finding (GAAP) heuristic. The GAAP strategy proposed a heuristic algorithm which aims to find the path of the AUV so that the value of information of the data delivered to sink nodes is maximized. It showed that the GAAP strategy delivers much more value of information than Random Selection (RS), Lawn Mower (LM) and Traveling Sales Man (TSP) strategies do. Nevertheless, the advantage of the GAAP strategy over the TSP strategy decreases with the network size which enables TSP strategy to collect more packets, and the average end-to-end delay of GAAP strategy is higher than TSP strategy.

Meanwhile, many energy-efficient forwarding methods are devised to prolong the network lifetime. Hu et al. [7] proposed a *Q*-Learning-based Energy-Efficient and Lifetime-Aware Routing (QELAR) Protocol for Underwater Sensor Networks. QELAR adopted *Q*-Learning algorithm which defines the residual energy of sensor nodes as the reward function. Therefore, in QELAR protocol, sensor nodes select the node with the most residual energy as the relay node, thus the network lifetime can be prolonged. However, QELAR did not constrain the end-to-end delay, which resulted in longer delay when the number of sensor nodes was increasing. Coutinho et al. [8] devised an Energy Balancing Routing (EnOR) Protocol for Underwater Sensor Networks. The EnOR protocol adopted the idea of balancing the energy consumption among neighboring nodes in the forward set by rotating the priority of them so as to extend the network lifetime. However, a large candidate set results in high delay because the link quality of the high priority nodes is usually low given the long distance between the sender and the high priority nodes. In addition, Jin et al. [9] proposed a *Q*-Learning-based Delay-Aware Routing (QDAR) Algorithm to Extend the Lifetime of Underwater Sensor Networks. It took both timeliness and energy efficiency into account by defining delay-related cost and energy-related cost.

Moreover, several studies of mobility of sensor nodes dealt with topology changes due to node mobility. For instance, Liu et al. [10] proposed an Opportunistic Forwarding Algorithm based on Irregualar Mobility (OFAIM). OFAIM aims to maximize the network delivery ratio of UWSNs in a 3-D mobility model due to irregular movement. However, there are only sensor nodes but no sink nodes in the scenario of OFAIM, and no descriptions of how the data will be retrieved from underwater sensors.

Additionally, there are some approaches that reduce energy consumption in consideration of node mobility. Forster et al. [11] proposed a Role-Free Clustering with *Q*-Learning (CLIQUE) for WSNs, which determines the selection of cluster heads without control overhead. The number of hops to reach mobile sink nodes and the residual energy of sensor nodes are jointly adopted as the reward function, thus enhancing the energy efficiency. However, CLIQUE assumed that sensor nodes uniformly disseminate data without consideration of the limited storage of sensor nodes. Webster et al. [12] invented a clustering protocol for UWSNs based on the mobility model proposed by Caruso et al. [13], which aims to minimize the overall energy consumption.

We distinguish our work from the above-mentioned ones as follows. Existing studies dealt with either energy consumption or timeliness of data forwarding in stationary topology, or simply considered energy consumption in dynamic topologies. None of these studies jointly considered all of them. Therefore, we propose a data forwarding method in joint consideration of timeliness and energy efficiency in UWSNs with passive mobility.

## 3. Materials and Methods

### 3.1. Preliminaries and Notations

#### 3.1.1. System Model

**Figure 1:**
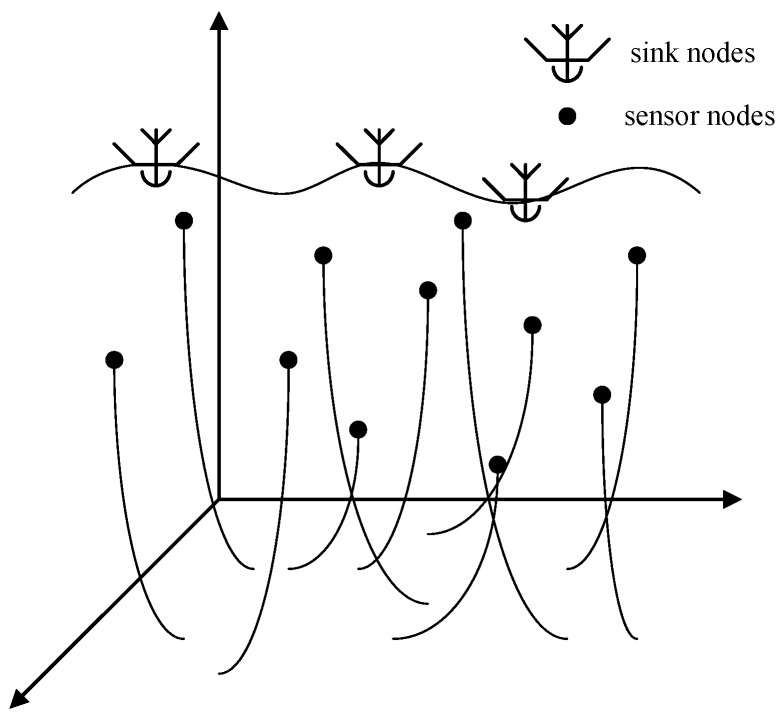
An Underwater Wireless Sensor Network (UWSN) with passive mobility.

The UWSN is represented by an undirected graph G(t)=(V,E(t)) at time slot *t*, where *V* is the set of sensor nodes and E(t) is the set of links between pairs of nodes within the communication range of each other at time slot *t*. As depicted in Figure 1, *N* sensor nodes are tethered to the water bottom via wires, and move passively due to internal currents or vortices.

The moving region is a semi-sphere with a radius of $R_{i}$ while the communication range of sensor $v_{i}$ is denoted by CR. $C_{i}\left(t\right)$ denotes the 3D-coordinate of $v_{i}\in V$ at time slot *t*, which is expressed as $\left(x_{i}\left(t\right),y_{i}\left(t\right),z_{i}\left(t\right)\right)$. If $|C_{i}\left(t\right)-C_{j}\left(t\right)|\leq CR$, then ${\left(v_{i},v_{j}\right)}_{t}\in E\left(t\right)$ is a bidirectional link and $v_{j}$ is a neighbor of $v_{i}$. H(i,t) denotes the set of neighbors of $v_{i}$ at time slot *t*.

Meanwhile, we have *M* sink nodes deployed on the water surface and the set of sink nodes are denoted by *S*. Additionally, S(i,t) denotes the set of sink nodes which are within the communication range of $v_{i}$. Sink node $s_{m}\in S$ is mounted on an autonomous draft so that $s_{m}$ can hold its position. In addition, they are equipped with acoustic modems for sensors and RF modems for satellites, along with access to GPS localization. Data packets are periodically generated and $P_{i,t}$ denotes the set of packets in $v_{i}$ at time slot *t* while $p_{i,t}$ denotes the *p*-th packet in $v_{i}$ at time slot *t*. Sensor nodes learn to forward packets to sink nodes in terms of Value of Information and the energy consumption of sensor nodes. Packets are supposed to be received by sink nodes via multi-hop relays.

In order to leverage the broadcast property of the wireless channel, each packet is acknowledged implicitly. Specifically, after transmitting a packet, the sender starts listening to the channel. If it overhears the packet being retransmitted within a certain period of time, the packet is regarded as successfully transmitted; otherwise, the packet is considered to be lost and the sensor node will learn to retransmit it, which will be described in detail in Section 4.

#### 3.1.2. Underwater Movement Model

**Figure 2:**
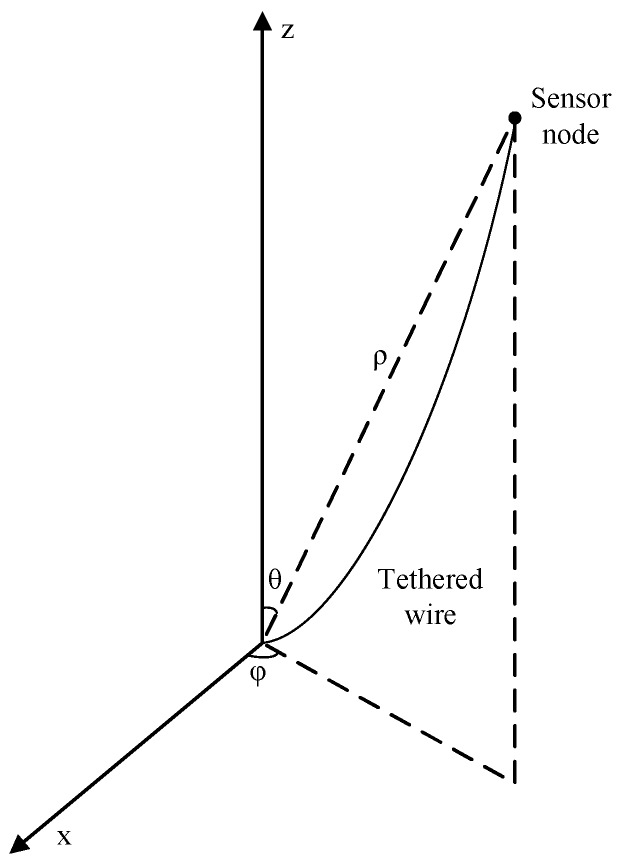
Movement model of sensor nodes.

The movement model is shown in Figure 2. We assume that the moving speed of $v_{i}$ is denoted as $SP_{i}\left(t\right)$ obeys the normal distribution $N(\mu_{1},\sigma_{1}^{2})$ and its actual value range is $\left(0,2\mu_{1}\right)$. $\left(d\theta_{i}\left(t\right),d\phi_{i}\left(t\right)\right)$ denotes the movement direction of $V_{i}$ at time slot *t*, where $d\theta_{i}\left(t\right)$ and $d\phi_{i}\left(t\right)$ obey uniform distributions U(0,π) and U(0,2π), respectively. The next location of $v_{i}$ from its current location $C_{i}\left(t\right)=\left(x_{i}\left(t\right),y_{i}\left(t\right),z_{i}\left(t\right)\right)$ will be:

(1) $$C_{i}\left(t+1\right)=\left\{\begin{array}{c} x_{i}\left(t\right)+SP_{i}\left(t\right)\sin d\theta_{i}\left(t\right)\cos d\phi_{i}\left(t\right)\\ y_{i}\left(t\right)+SP_{i}\left(t\right)\sin d\theta_{i}\left(t\right)\sin d\phi_{i}\left(t\right)\\ z_{i}\left(t\right)+SP_{i}\left(t\right)\cos d\theta_{i}\left(t\right) \end{array}\right.$$

when $|C_{i}\left(t\;+\;1\right)|>R_{i}$, where $R_{i}$ denotes the length of the tethering wire of $v_{i}$, the node is held still by its tethered wire and $C_{i}\left(t+1\right)$ can be written as $\left(R_{i},\theta_{i}\left(t\right)+d\theta i\left(t\right),\phi_{i}\left(t\right)+d\phi i\left(t\right)\right)$ in spherical coordinates. Otherwise, $C_{i}\left(t+1\right)$ is defined by Formula (1).

#### 3.1.3. Value of Information

Immediate detection of regions of interest in early stage can provide sufficient time to take corresponding actions. Hence, we adopt the concept of value of information which evaluates information in terms of timeliness. Hence, the later a packet is forwarded to the sink, the lower its value is. Therefore, the VoI of a packet can be expressed as Equation (2),

(2) $$VoI\left(p_{i,t}\right)=ke^{-\alpha t_{l}},\;t_{l}\in \left[0,TTL\right]$$

where $p_{i,t}$ represents the *p*-th packet in $v_{i}$ at time slot *t*, $t_{l}$ indicates the living time duration of packet $p_{i,t}$ since it is generated, α is the decay factor, *k* is the discount coefficient and TTL is the maximum life of the packet, i.e., time to live.

$VoI(p_{i,t})$ is a key factor of the decision making of a sensor node as to which packet should be relayed. If the living duration of a packet approaches its TTL, it will be discarded immediately.

#### 3.1.4. Energy Consumption

Each sensor node has its battery capacity, and with adjustable transmission power. The energy consumption of a sensor mainly includes the energy consumed on the sensor module, its processor module and its communication module, among which the communication module consumes the most energy. Hence, the energy consumption of a sensor node can be approximated by the communication energy consumption while ignoring its other energy consumptions. According to the typical model of energy consumption of free-space spherical wave, the energy consumption of a sensor node is:

(3) $$\left\{\begin{array}{c} E_{c}=E_{Rx}+E_{Tx}\\ E_{Rx}=pl\cdot e_{s}\\ E_{Tx}=pl\cdot \left(e_{s}+e_{r}d^{2}\right) \end{array}\right.$$

where pl is the data volume that a sensor node receives or transmits, in bit; $e_{s}$ is the circuit energy consumption of emitting or receiving per bit data, in J/bit; $e_{r}$ is the minimum energy of signal per bit that can be received by sensor nodes or sink nodes successfully, in $J/(\mathrm{bit}\cdot m^{2})$; *d* is the communication distance, in meter.

#### 3.1.5. Forwarding Orientation

In order to prolong the longevity of UWSNs, it is significant to adopt an energy-efficient forwarding method. Inspired by the murmuration of a swarm of swallows, Pearce et al. [14] proposed a biotic model, the Hybrid Projection Model, which defines the murmuration via two metrics: the opacity and the orientation.

**Figure 3:**
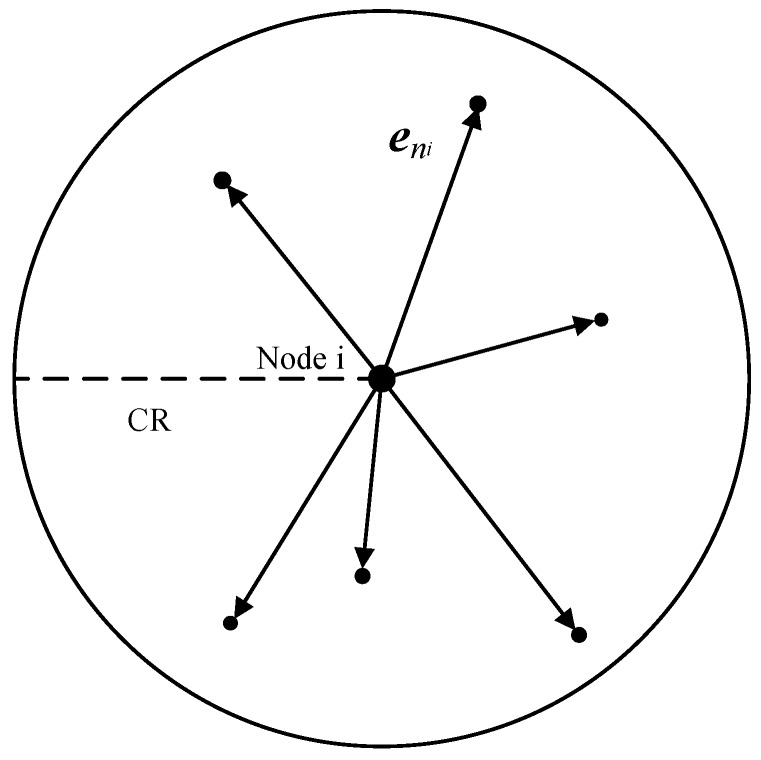
Orientation of sensor nodes.

As can be seen in Figure 3, the orientation is mathematically defined as average accumulation of vectors created by the neighbors of a node, which can be calculated by Equation (4),

(4) $$e_{ori}\left(i,t\right)=\frac{1}{\left|H(i,t)\right|}\underset{|H(i,t)|\;\mathrm{neighbor}\;\mathrm{vectors}}{\underset{︸}{\left(v_{1}\left(i,t\right)+v_{2}\left(i,t\right)+\ldots +v_{j}\left(i,t\right)+\ldots +v_{\left|H(i,t)\right|}\left(i,t\right)\right)}},\;v_{j}\left(i,t\right)\in H(i,t)$$

where $e_{ori}\left(i,t\right)\in R^{3}$ denotes the vector of orientation, |H(i,t)| is the number of neighbors of $v_{i}$ within its communication range and $v_{j}\left(i,t\right)\in R^{3}$ denotes the vector from $v_{i}$ to its *j*-th neighbor $v_{j}$ at time slot *t*. The orientation can be acquired locally via the Received Signal Strength (RSS) and Arrival of Angle (AoA) of the broadcasting packets from neighborhood.

The length of $e_{ori}\left(i,t\right)$ denotes the absolute value of the orientation and the orientation direction is denoted by the direction of $e_{ori}\left(i,t\right)$. Nodes with large orientation values are generally located on the edge of a neighborhood. Otherwise, they are near the centers of their neighborhoods and nodes with lower orientation values are more likely to be the relay node. It has been proved that determining the forwarding direction via orientation metric is energy-efficient [12]. Moreover, there is no requirement for localization when using the orientation metric, which is very suitable for underwater sensors due to their inaccessibility to GPS signals. Therefore, we adopt the orientation metric to determine data forwarding direction.

### 3.2. Problem Definition

Given a UWSN G(t)=(V,E(t)) at time slot *t*. As mentioned above, we ascertain the objective as minimizing the energy consumption of data forwarding with maximal Value of information within a given monitoring duration *T*. Therefore, we aim to solve the problem of data forwarding by jointly considering timeliness and energy consumption.

(5) $$\min\sum_{t=0}^{T}\sum_{i=0}^{N}E_{c}\left(i,t\right)$$

$$\begin{array}{c} s.t. \end{array}$$

(6) $$\begin{array}{c} p_{i,t}^{*}=\arg\max_{p_{i,t}\in P_{i,t}}VoI\left(p_{i,t}\right) \end{array}$$

(7) $$\begin{array}{c} \forall v_{i}\in V,t\leq T,\;E_{r}\left(i,t\right)\geq 0 \end{array}$$

(8) $$\begin{array}{c} \forall v_{i}\in V,p_{i,t}\in P_{i,t},\;t_{l}\leq TTL \end{array}$$

(9) $$\begin{array}{c} \forall v_{i}\in V,t\leq T,\;\left|C_{i}\left(t+1\right)\right|\leq R_{i} \end{array}$$

As shown in Equation (6), $p_{i,t}^{*}$ represents the candidate packet which has the highest value of information in $v_{i}$ at time slot *t*. Furthermore, if $v_{i}$ is able to forward data to any neighbor at time slot *t*, $p_{i,t}^{*}$ will be delivered. In Equation (7), each sensor node has limited energy and is out of use when its residual energy hits the bottom at 0. The living time of packets cannot exceed the maximum living duration TTL as shown in Equation (8). In Equation (9), the moving range of each sensor node is limited to the length of its tethered wire $R_{i}$.

### 3.3. Data Forwarding Method

In our scenario, the sensor nodes are dynamically moving due to water flow. In addition, the environment and neighborhood topology of each sensor node keep changing. We adopt a reinforcement learning-based method by which sensor nodes can distributively learn from the changing environments to forward data. This section describes the data forwarding method in detail. Specifically, we present the learning model, the learning method to choose a relay and the algorithm for packet forwarding.

#### 3.3.1. Data Forwarding Procedure

The procedure of data forwarding mainly contains the following three stages, as can be seen in Algorithm 1.

(1) In the beginning of each time slot, each sensor node and sink node broadcasts its beacon signal, e.g., the identifier, orientation and residual energy. Therefore, each sensor node knows its neighbors.
(2) When $v_{i}$ hears the beacon signal from $s_{m}$, it adds $s_{m}$ to the set of its available sink nodes S(i,t). Similarly, if $v_{i}$ can hear the beacon signal of sensor node $v_{j}$, $v_{i}$ will add $v_{j}$ to the set of its neighbors H(i,t). Additionally, the distance and orientation of each neighbor or reachable sink node can be acquired locally via the Received Signal Strength (RSS) and Arrival of Angle (AoA) of the beacon signal, respectively. If $v_{i}$ cannot hear from any sink nodes or sensor nodes, $v_{i}$ will wait until the next time slot coming.
(3) Sensor node $v_{i}$ selects the reachable sink node or next relay node by the algorithm RelaySelect which performs a learned choice of a relay node. The RelaySelect algorithm will be introduced in detail in the third subsection.

**Algorithm 1** DataForwarding(t,C(t)).
1:
**for each** $v_{i}\in V$ **do**
2: S(i,t)=∅3: H(i,t)=∅4:
**end for**
5:
**for each** $s_{m}\in S$ **do**
6: $s_{m}$ broadcasts its beacon signal7: **for each** $v_{i}\in V$ **do**8:  **if** $v_{i}$ can hear $s_{m}$ **then**9:   $S\left(i,t\right)=S\left(i,t\right)\cup \left\{s_{m}\right\}$10:  **end if**11: **end for**12:
**end for**
13:
**for each** $v_{i}\in V$ **do**
14: $v_{i}$ broadcasts its beacon signal15:
**end for**
16:
**for each** $v_{i}\in V$ **do**
17: **for each** $v_{i}\in V,j\neq i$ **do**18:  **if** $v_{i}$ can hear $v_{j}$ **then**19:   $H\left(i,t\right)=H\left(i,t\right)\cup \left\{v_{j}\right\}$20:  **end if**21: **end for**22:
**end for**
23:
**for each** $v_{i}\in V$ **do**
24: $a_{i}\left(t\right)=RelaySelect\left(S\left(i,t\right),H\left(i,t\right),P\left(i,t\right)\right)$25:
**end for**



#### 3.3.2. *Q*-Learning Model

*Q*-Learning is a model-free reinforcement learning technique, based on agents taking actions and receiving rewards from the environment in response to actions [11]. Each action is evaluated a *Q*-value due to its fitness. In the learning process, the agent calculates the reward of each potential action and updates the *Q*-value by which the real action can be determined. *Q*-Learning has been widely adopted in wireless ad hoc communications. The main challenge is the modeling of the *Q*-Learning process and the definition of *Q*-values.

Given the set $X=\{x_{1},x_{2},\ldots ,x_{t},\ldots ,x_{T}\}$ of states of an agent, a reward $r_{t}\left(a_{t}\right)$ is received in state $x_{t}$ after the agent takes action $a_{t}\in A$ at time slot *t*.

To evaluate how good an action is at a state, the *Q*-value of action $a_{t}$ at time slot *t*, $Q(x_{t},a_{t})$, is updated as follows:

(10) $$Q\left(x_{t},a_{t}\right)=r_{t}\left(a_{t}\right)+\gamma \sum_{x_{t+1}\in X}P_{x_{t}\to x_{t+1}}^{a_{t}}Q(x_{t+1},a_{t+1})$$

where $r_{t}\left(a_{t}\right)$ is the reward of taking action $a_{t}$ at time slot *t*, $Q(x_{t+1},a_{t+1})$ is the expected fitness at time slot (t+1), γ is the learning discount factor and $P_{x_{t}\to x_{t+1}}^{a}$ represents the transition probability from state $x_{t}$ to $x_{t+1}$.

In order to determine the optimal action, the action with the highest *Q*-value from state $x_{t}$ to $x_{t+1}$ at time slot *t* can be acquired as follows:

(11) $$a_{t}^{*}=\arg\max_{a_{t}\in A}Q(x_{t},a_{t})$$

For each state $x_{t}\in X$, the optimal action $a_{t}^{*}$ can be greedily acquired by updating the *Q*-value.

#### 3.3.3. Learning to Forward

If $v_{i}$ transmits a packet to a relay node or a sink node, the state of $v_{i}$ at time slot (t+1) turns to 1, $x_{t+1}=1$. Otherwise, $x_{t+1}=0$. The action $a_{t}$ in our scenario is $a_{i}\left(t\right)=\left(p_{i,t},v_{j}\right)$ which denotes the action of $v_{i}$ forwarding packet $p_{i,t}$ to $v_{j}$. Then, the reward of of taking action $a_{t}$ to next state $x_{t+1}$ is described as $r(p_{i,t},v_{j})$. Lastly, the *Q*-value is updated to $Q(p_{i,t},v_{j})$ which indicates the fitness of $v_{i}$ forwarding packet $p_{i,t}$ to $v_{j}$ at time slot *t*.

In our data forwarding scenario, each sensor node is an independent learning agent and actions are options of a relay node or a sink node within its communication range. The following describes details of the model solution, including time, actions, transmission probabilities, rewards, and *Q*-values.

***Agents*** Agents are underwater sensor nodes.

***Time*** A $v_{i}$ handling a packet *p* is associated with a time slot t∈{0,1,2,3,…,T} defined by the sequential number of time slots.

***Actions*** Actions refer to the joint selection of a packet in the node’s cache and of a relay node in its neighborhood. The set $A_{i}\left(t\right)$ of available actions is $A=\{a_{i}\left(t\right)=\left(p_{i,t},v_{j}\right)|p_{i,t}\in P_{i,t},v_{j}\in H\left(i,t\right)\}$, where $a_{i}\left(t\right)=\left(p_{i,t},v_{j}\right)$ is the action of forwarding packet $p_{i,t}$ to relay node $v_{j}$.

***Transmission Probabilities*** Denote the probability of transmission from $v_{i}$ to $v_{j}$ at time slot *t* as $Pr_{i,j}\left(t\right)$. Meanwhile, the transmission probability from the current relay node $v_{j}$ to the next potential relay node $v_{k}$ is denoted by $Pr_{j,k}\left(t+1\right)$. $Pr_{i,j}\left(t\right)$ is computed by $v_{i}$ while $Pr_{j,k}\left(t+1\right)$ is computed by $v_{j}$ and sent to $v_{i}$ in the header of the broadcast packet in each round. The transmission probabilities can be calculated via the orientation metric by Equation (12), as follows.

(12) $$\left\{\begin{array}{c} Pr_{i,j}\left(t\right)=1-\frac{1}{\pi }\arccos\frac{e_{ori}\left(i,t\right)v_{n_{i}}\left(i,t\right)}{|e_{ori}\left(i,t\right)||v_{n_{i}}\left(i,t\right)|}\\ Pr_{j,k}\left(t+1\right)=1-\frac{1}{\pi }\arccos\frac{e_{ori}\left(j,t\right)v_{n_{j}}\left(j,t\right)}{|e_{ori}\left(j,t\right)||v_{n_{j}}\left(j,t\right)|} \end{array}\right.$$

Note that $Pr_{j,k}\left(t+1\right)$ is the prediction from the current time slot *t* because the topology at time slot (t+1) cannot be ascertained yet due to the node mobility.

***Rewards*** The rewards mainly consist of two aspects, energy consumption and VoI, as shown in Equation (13),

(13) $$r\left(p_{i,t},v_{j}\right)=VoI\left(p_{i,t}\right)\cdot E_{r}\left(i,t\right)$$

where $r(p_{i,t},v_{j})$ represents the reward of $v_{i}$ transmitting packet $p_{i,t}$ to $v_{j}$, $VoI(p_{i,t})$ denotes the VoI of packet $p_{i,t}$, and $E_{r}\left(i,t\right)$ represents the residual energy of $v_{i}$ after transmission, at time slot *t*.

***Q-values** Q*-values represent the goodness of actions and agents aim to learn the actual fitness of potential actions. We initialize the *Q*-values as shown in Equation (14),

(14) $$Q\left(p_{i,t},v_{j}\right)=VoI\left(p_{i,t}\right)\cdot E_{r}\left(i,t\right)$$

where $Q(p_{i,t},v_{j})$ refers to the *Q*-value of $v_{i}$ in response to the action of choosing $v_{j}$ as the relay node, $VoI(p_{i,t})$ denotes the VoI of packet $p_{i,t}$ to be transmitted, and $E_{r}\left(i,t\right)$ represents the residual energy of $v_{i}$, in the beginning.

Algorithm 2 describes the learning process of $v_{i}\in V$ in each time slot as well as the corresponding determination of the packet to forward and its relay node.

If sink node $s_{m}\in S$ is within the transmission range of $v_{i}$, $v_{i}$ transmits the packet with the largest VoI in its cache to $s_{m}$ directly. Otherwise, to identify an optimal forwarding decision, $v_{i}$ learns the value of function $Q(p_{i,t},v_{j})$ and updates the *Q*-value. Based on this value $v_{i}$ determines the optimal forwarding action $a_{i}\left(t\right)=\left(p_{i,t},v_{j}\right)$. Each node starts with no knowledge of its surrounding environment. Broadcasting and listening in neighborhood, sensor nodes iteratively acquire and update their knowledge over time. Function $r(p_{j,t+1},v_{k})$ in Equation (15) is approximated via Equation (13) based on the localization and neighborhood at time slot *t* The *Q*-values can be updated as shown in Equation (15),

(15) $$Q\left(p_{i,t},v_{j}\right)=r\left(p_{i,t},v_{j}\right)+\gamma \sum_{k\in H(j,t),k\neq j,k\neq i}Pr_{j,k}\left(t+1\right)r\left(p_{j,t+1},v_{k}\right)$$

where $r(p_{j,t+1},v_{k})$ is the reward of $v_{j}$ transmitting packet $p_{j,t+1}$ to $v_{k}$ at time slot (t+1), and $r(p_{j,t+1},v_{k})$ is approximated via Equation (13) based on the localization and neighborhood at time slot *t*. Additionally, $Pr_{j,k}\left(t+1\right)$ represents the probability of transmission from $v_{j}$ to $v_{k}$ and γ is the learning factor. In the learning process, sensor nodes calculate the reward of each potential relay node and update the *Q*-value. Finally, sensor nodes acquire the *Q*-table by which the most appropriate relay node can be determined.

**Algorithm 2** RelaySelect(S(i,t),H(i,t),P(i,t)).
1:
**for each** $v_{i}\in V$ **do**
2: **if** $\exists s_{m}\in S\left(i,t\right)$ **then**3:  $p_{i,t}=\arg\max_{p_{i,t}\in P_{i,t}}VoI\left(p_{i,t}\right)$4:  $a_{i}\left(t\right)=\left(p_{i,t},s_{m}\right)$5: **else**6:  **for each** $p_{i,t}\in P_{i,t}$ **do**7:   **for each** $v_{j}\in H\left(i,t\right)$ **do**8:    **for each** $v_{k}\in H\left(j,t\right)$ **and** k≠i **do**9:     $Q\left(p_{i,t},v_{j}\right)=r\left(p_{i,t},j\right)+\gamma \sum_{k\in H(j,t),k\neq j,k\neq i}Pr_{j,k}\left(t+1\right)r\left(p_{j,t+1},k\right)$10:    **end for**11:   **end for**12:  **end for**13:  $\left(p_{i,t},v_{j}\right)=\arg\max_{a_{i}\left(t\right)\in A}Q\left(p_{i,t},v_{j}\right)$14:  $a_{i}\left(t\right)=\left(p_{i,t},v_{j}\right)$15: **end if**16: **return** $a_{i}\left(t\right)$17:
**end for**



In our method, each sensor node has to ascertain its neighborhood and then selects the relay node in its neighborhood. Specifically, we have to execute two rounds of calculation for each sensor node in each time slot: (1) the determination of neighbor nodes within the sensor’s communication range; (2) the selection of the neighbor node with highest *Q*-value. In the first round of calculation, it takes a complexity of $O(\frac{N(N-1)}{2})$ to calculate the distances between sensor nodes. In the second round, the complexity depends on the size of the neighborhood of sensor nodes. In the most complicated case, all the sensor nodes in the same neighborhood, i.e., $\forall j\neq i,v_{j}\in H_{i}$, we need to calculate (N−1) times of *Q*-value of the neighbor nodes of $v_{i}$. Therefore, it takes a complexity of O(N(N−1)) at most to select relay nodes of all the sensor nodes. Since the number of time slots is constant, the complexity of our method can be ascertained as $O(N^{2})$.

## 4. Results

In this section, we evaluate the performance of our proposed method compared with two well-known routing protocols: (i) QELAR, a machine learning-based protocol designed for minimizing and balancing node energy consumption [7]; (ii) DBR, a data forwarding method for UWSNs based on the depth of the sender [15]. It is worth mentioning that we use the total residual energy of sensor nodes, Value of Information and the ratio of packet delivery to sink nodes as the main metrics of performance evaluation.

### 4.1. Experimental Setup

The region of interests cover a space of 1000 m × 1000 m × 1000 m. We assume that the anchors are randomly deployed at the bottom and the length of tethering wires are also randomly generated, while the sink nodes are stationary at (333, 333, 1000) m and (666, 666, 1000) m. We consider UWSNs with different sizes of 10 and 100 sensor nodes, respectively. The sensors use Orthogonal Frequency-division Multiplexing (OFDM) modulation which allows simultaneous transmission from several users.

The simulation parameters are shown in Table 1. Each sensor node has a communication range of 300 m with initial energy of 100 J. The packets are set to the length of 1000 bit with the TTL of 10 time slots. Sensor nodes move passively at a maximum speed of 100 m per time slot. The coefficient of energy consumption $e_{s}$ and $e_{r}$ are set to $5\times 10^{-8}\;J/\mathrm{bit}$ and $10^{-8}\;J/(\mathrm{bit}\cdot m^{2})$, respectively. The decaying factor of VoI, i.e., α, is set as 0.5, while the learning discount factor γ is set as 1, which speeds up the learning rate. All simulation results are acquired with runs of 100 times.

### 4.2. Simulation Metrics

Data forwarding performance is assessed through the following three metrics.

***Value of Information*** defined as the VoI of packets acquired by the sink nodes within the monitoring duration.

***Residual Energy*** defined as the total residual energy of sensor nodes within the monitoring duration.

***Packet Delivery Ratio*** defined as the fraction of packets received by the sink nodes within the monitoring duration.

### 4.3. Simulation Results

In this section, we illustrate the results from simulations. All results are obtained by averaging over 100 simulation times.

**Figure 4:**
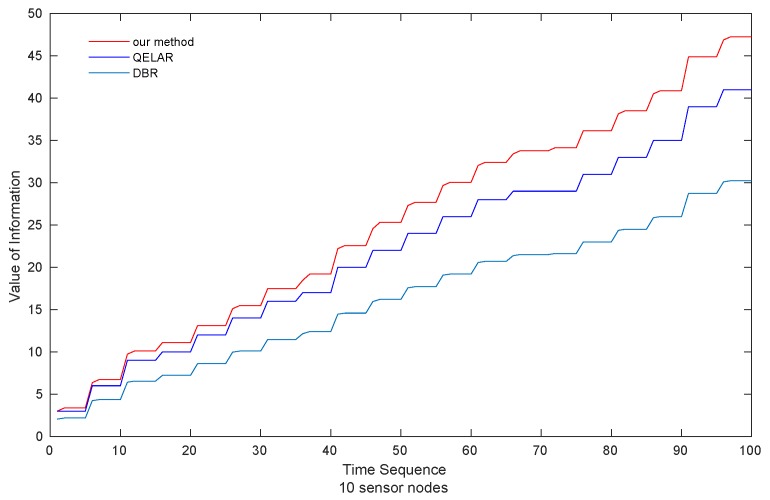
Value of Information obtained by sink nodes (10 sensor nodes).

(1) ***Value of Information*** As can be seen in Figure 4, the value of information acquired by sink nodes in the scenario of 10 sensor nodes is presented. Our method gains the highest VoI, 14.63% and 51.61% higher than QELAR and DBR, respectively. QELAR comes in the second place while DBR obtains the lowest VoI among the three methods.

**Figure 5:**
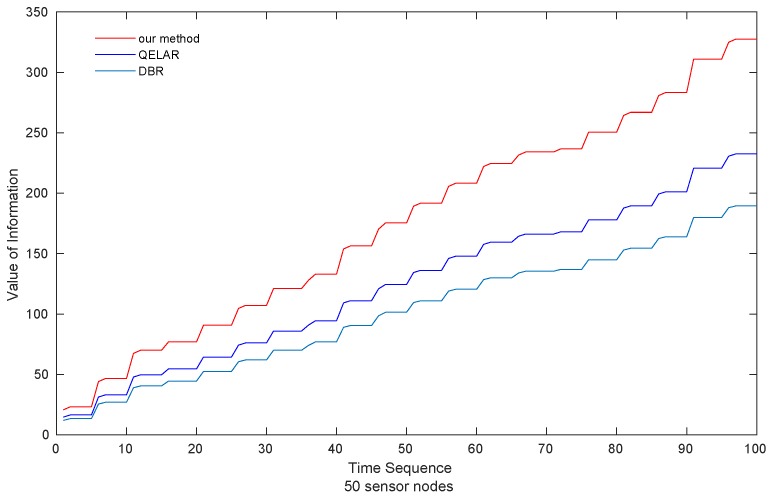
Value of Information obtained by sink nodes (50 sensor nodes).

Moreover, as shown in Figure 5, the VoI acquired by our method performs better as the network size increases, which is 43.48% and 83.35% higher than QELAR and DBR, respectively. When forwarding data, QELAR and DBR choose the earliest packet in the cache. Not surprisingly, DBR achieves the lowest VoI because the forwarding decision of DBR depends on the accessibility of neighbors with smaller depths. Specifically, compared with QELAR and our method, sensor nodes have to wait longer for the qualified neighbors, which leads to more decay of the VoI of packets. Our proposed method performs the highest VoI, because our method explicitly takes VoI into account in its reward function (Section 4), which leads to the choice of the packet with largest VoI in the sensor cache.

**Figure 6:**
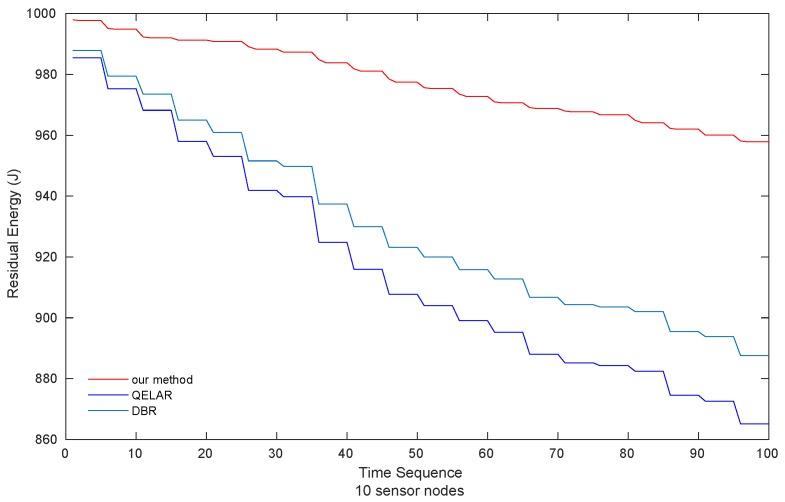
Residual energy of sensor nodes (10 sensor nodes).

(2) ***Residual Energy*** The results of residual energy of QELAR, DBR and our method with 10 sensor nodes is indicated in Figure 6. The residual energy of QELAR is the lowest while our method consumes the smallest energy among the three methods. More specifically, our method consumes 31.21% and 37.26% of the energy consumed by QELAR and DBR, respectively.

**Figure 7:**
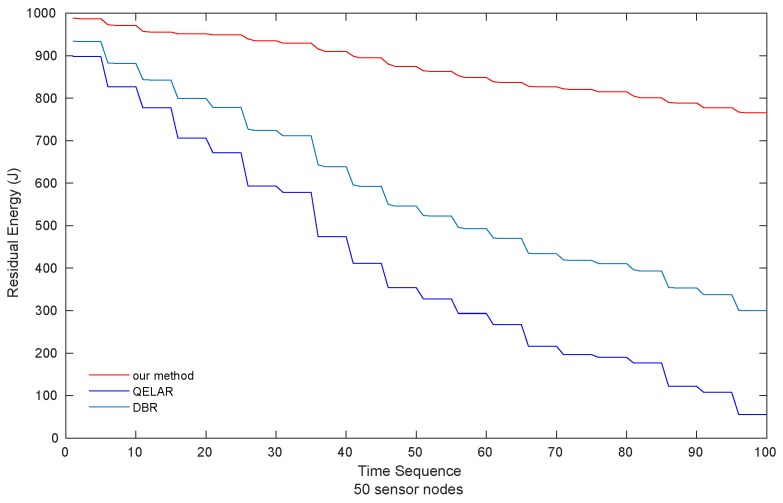
Residual energy of sensor nodes (50 sensor nodes).

As shown in Figure 7, our method still consumes the least energy among the three methods when the network size increases, only 24.79% and 31.43% of the energy consumption of QELAR and DBR, respectively. That is mainly because by choosing packets and relay nodes smartly, our method achieves excellent performance in energy consumption. Our method always selects the latest packets in the cache while QELAR always selects the earliest packets. Moreover, in our method, earlier packets may have been discarded due to TTL constraint when the latter packets are forwarded, which leads to the avoidance of forwarding too many early packets in the cache, compared with QELAR. Therefore, the energy consumption of our method is much lower than that of QELAR.

(3) ***Packet Delivery Ratio*** The packet delivery ratio (PDR) of QELAR, DBR and our method can be seen in Table 2. DBR achieves higher PDR than other two methods in both scenarios. Because packets are forwarded towards sensor nodes with less depths, the packets are either staying in a sensor node or approaching the water surface, which prevents the packets from being forwarded repeatedly between several sensor nodes and trapped in a certain region. Therefore, DBR decreases the repeating forwarding between sensor nodes and increases the PDR. The PDR to sink nodes of our method in scenarios of 10 and 50 sensor nodes are 66.36% and 71.64%, respectively. Our method achieves a PDR slightly lower than QELAR does, mainly because more packets with earlier generation time in the cache are discarded due to the maximum living duration.

## 5. Discussion and Conclusions

In this paper, we proposed the data forwarding method in joint consideration of VoI of packets and energy consumption, with passive mobility of sensors in UWSNs. We explicitly take both VoI and energy consumption into account in its reward function, thus reducing the energy consumption as well as enhancing the timeliness of data forwarding in UWSNs. In our method, the *Q*-value of the same sensor node can be different along the time, thus avoiding the same node acting as a relay node until the depletion of its battery. Meanwhile, packets with larger value of information have higher priority to be transmitted so as to realize better timeliness. Although the packet delivery ratio of our method is relatively lower, our proposed method achieves much higher timeliness and consumes less energy than DBR and QELAR in the circumstance of dynamical topology change due to the passive mobility of sensor nodes. Given that the timeliness and energy consumption were more significant than the delivery ratio in our scenario, our method enhances the performance of UWSNs. In our scenario, the sink nodes are stationary and the performance of data collection may be different if the sink nodes are moving on the surface of the detection region. As a future work, we will study how the movement of sink nodes can influence the data collection of UWSNs. Additionally, recent studies of harvesting ambient energy of UWSNs has drawn large attention. For instance, the kinetic energy of underwater currents can be harvested to prolong the lifetime of UWSNs. Therefore, we intend to carry out the research of energy harvesting-aware data forwarding in UWSNs with passive mobility in the future.

## Figures and Tables

**Table 1:** Simulation Parameters.

Name	Value
CR	300 m
pl	1000 bit
$E_{ini}$	100 J
$e_{s}$	$5\times 10^{-8}$ J/bit
$e_{r}$	$10^{-8}\;J/(\mathrm{bit}\cdot m^{2})$
SP	100 m per time slot
TTL	10 time slots
α	0.5
*k*	1
γ	1

**Table 2:** Packet Delivery Ratio.

PDR()	DBR	QELAR	Our Method
PDR(10)	79.47%	70.25%	66.36%
PDR(50)	93.12%	76.86%	71.64%
